# Supplementary material for: How Has the Hazard to Humans of Microorganisms Found in Atmospheric Aerosol in the South of Western Siberia Changed over 10 Years?
Source: Int J Environ Res Public Health. 2020 Mar 3;17(5):1651. doi: 10.3390/ijerph17051651 (PMC7084375; doi:10.3390/ijerph17051651)
Supplement: Supplementary file 1 [file ijerph-17-01651-s001.zip › List of descriptions.docx]

List of descriptions

The following descriptions are used in the cells of Tables S1 - S13.

The cell marked with : the characteristic was not determined.

Isolates marked with are yeasts; unmarked cells in these tables are bacteria.

Isolates cultured at 6 - 10 ºС are marked .

The maximum index values for each year are marked .

The maximum index values for sessions 2006 - 2008 and 2012 - 2016 are marked .

Indices average values for each year and its standard deviations are marked with .

Indices average values for sessions 2006 - 2008 and 2012 - 2016 and its standard deviations are marked with .

Missed data in Table S13 and calculations using these data are marked with .

All tables contain the calculations of integral indices for every isolates and calculation of average and Standard Error values for each year of investigation. Some characteristics that are needed to the calculations of integral indices may be not measured due to different reasons. In this case missed characteristics are replaced by its average values for the year of measurements.

The Table S13 contains Figures also that represent Tables 1 – 4 in the text.
